# Study Protocol for a Randomised Controlled Trial on Pulmonary Metastasectomy vs. Standard of Care in Colorectal Cancer Patients With ≥ 3 Lung Metastases (PUCC-Trial)

**DOI:** 10.3389/fonc.2022.913896

**Published:** 2022-07-11

**Authors:** Severin Schmid, Heiko Becker, Ralph Fritsch, Johannes Bausch, Natalie Hunter, Carolin Jenkner, Mohamed Hassan, Bernward Passlick

**Affiliations:** ^1^ Department of Thoracic Surgery, Medical Center – University of Freiburg, Faculty of Medicine, University of Freiburg, Freiburg, Germany; ^2^ Department of Medicine I, Medical Center - University of Freiburg, Faculty of Medicine, University of Freiburg, Freiburg, Germany; ^3^ Department of Medical Oncology and Hematology – University Hospital of Zurich, Zurich, Switzerland; ^4^ Clinical Trials Unit, Medical Center, Faculty of Medicine, University of Freiburg, Freiburg, Germany

**Keywords:** colorectal (colon) cancer, pulmonary metastasectomy, overall survival (OS), quality of life, metastasis

## Abstract

This is a multicentre prospective randomised controlled trial for patients with 3 or more resectable pulmonary metastases from colorectal carcinoma. The study investigates the effects of pulmonary metastasectomy in addition to standard medical treatment in comparison to standard medical treatment plus possible local ablative measures such as SBRT. This trial is intended to demonstrate an overall survival difference in the group undergoing pulmonary metastasectomy. Further secondary and exploratory endpoints include quality of life (EORTC QLQ-C30, QLQ-CR29 and QLQ-LC29 questionnaires), progression-free survival and impact of mutational status. Due to the heterogeneity and complexity of the disease and treatment trajectories in metastasised colorectal cancer, well powered trials have been very challenging to design and execute. The goal of this study is to create a setting which allows treatment as close to the real life conditions as possible but under well standardised conditions. Based on previous trials, in which patient recruitment in the given setting hindered successful study completion, we decided to (1) restrict inclusion to patients with 3 or more metastases (since in case of lesser, surgery will probably be the preferred option) and (2) allow for real world standard of care (SOC) treatment options before and after randomisation including watchful waiting (as opposed to a predefined treatment protocol) and (3) possibility that patient can receive SOC externally (to reduce patient burden). Moreover, we chose to stipulate 12 weeks of systemic treatment prior to possible resection to further standardize treatment response and disease course over a certain period of time. Hence, included patients will be in the disease state of oligopersistence rather than primary oligometastatic. The trial was registered in the German Clinical Trials Register (DRKS-No.: DRKS00024727).

## Introduction

Pulmonary metastasectomy (PM) is a widely applied treatment for metastasised colorectal cancer (mCRC) based on findings from a vast abundance of retrospective trials (1–5). The only prospective trial on pulmonary mCRC has not shown any survival benefit for patients undergoing PM compared to systemic therapy only (6). Although the trial failed to reach its recruitment target and was thus underpowered the observed survival of 47% after 4 years in the control group is far better than expected and crucial when assuming a potential benefit from PM (7). Other prospective randomised trials have demonstrated a benefit for progression-free as well as overall survival by radical local ablative treatments in metastasised solid cancers including lung, breast, colorectal cancer (CRC) and others (8–11). Local ablative measures in these trials were either exclusively or mostly non-surgical consisting of stereotactic body radiation therapy (SBRT) and radiofrequency ablation. Moreover, the only trial exclusively including patients with hepatic metastases from CRC suffered, although randomised, from serious imbalances regarding number of metastases in the investigated groups (8).

Generally, the application of local ablative measures in metastasised cancers remains controversial. Some argue that systemic diseases should be treated as such and hence therapy should be confined to systemic treatment alone (12). Others believe that radical local measures result in survival advantages due to cytoreduction and removal of sites which are insufficiently treated by the medical treatment. Also, these sites could be capable of seeding new metastases. The significance of tumour cell release by secondary tumours for further metastasisation remains unclear, however, there is clinical and experimental evidence showing a beneficial effect of aggressive local ablative treatment in oligometastasis on further metastasisation (13–19)

Current guidelines of the European Society of Medical Oncology (ESMO) recommend resection of pulmonary metastases in cases in which R0-resection is feasible, however under consideration of relative contraindications based on the tumour biology as well as patient-related factors such as comorbidities and personal expectations (20).

The factors currently defining tumor biology include presence of a higher number of metastases, meta- vs. synchronicity of metastasisation and a short interval from diagnosis of the primary to first manifestation of metastasis [disease-free-interval (DFI)]. Due to the lack of strong evidence the interpretation of these relative contraindications is highly variable and the chosen treatment modalities depend largely on the treating institution and discipline. A benefit from surgical resection has never been proven in prospective trials even in patients with small numbers of metastases (1-3). Nevertheless, surgery in these patients is generally applied and considered treatment of choice in many countries. This is based primarily on retrospective data which shows a favourable prognosis in patients with completely resectable metastases, even if there are more than 3 lesions (2, 5). If surgical resection in comparison to the current standard of care proves superior in the study presented here, PM could be implicated as standard of care option also in patients with multiple metastases and thus help to improve long-term survival of these patients; on the other hand, a negative finding could result in abandoning the practice of PM at least in a selected cohort.

To our knowledge there is currently only one ongoing other multi-centric prospective randomised controlled trial on PM conducted by the MD Anderson Cancer Center in Texas, USA which started recruitment in July 2018 (NCT03599752). Patients are categorised into low and high risk before being randomised. Low risk patients are randomised to either PM + systemic treatment or PM alone. High-risk patients are randomised to either PM + systemic treatment or systemic treatment alone. Primary outcome measures are progression-free survival (PFS) in the low-risk group and overall survival (OS) in the high-risk group.

Although past prospective randomised trials either failed to reach the targeted recruitment numbers and/or suffered from patient heterogeneity we hope that further, carefully designed prospective trials, as the one we present here, can provide additional insight into the potential benefits of surgical removal of pulmonary metastases in patients with CRC.

## Methods and Analysis

### Design

This is a multicentre prospective randomised controlled trial for patients with 3 or more resectable pulmonary metastases from CRC. PUCC investigates the effects of pulmonary metastasectomy in addition to standard medical treatment in comparison to standard of care, i.e. medical treatment and/or alternative local ablative measures such as stereotactic body radiation therapy (SBRT).

This trial is intended to demonstrate an overall survival benefit in the group undergoing pulmonary metastasectomy. For the trial start a total of about 15 sites have agreed to participate in this multicentre trial. Then, if necessary more sites will be included and in case of recruitment failure, the respective sites will be replaced. The planned recruiting period is 2 years.

### Endpoints

The primary endpoint is overall survival (OS). OS is defined as the time from randomisation until death from any cause with censoring at the last date alive. The primary objective is to assess the effect of pulmonary metastasectomy compared to standard of care consisting of systemic therapy and possible SBRT where indicated on OS.

Secondary endpoints are

- Progression-free survival (PFS), defined as the time from randomisation until disease progression or death from any cause.
  - PFS assessment will be performed locally, usually in a multidisciplinary setting (e.g. tumour board) but at least by a radiologist and oncologist/thoracic surgeon.
  - Definitions of progressive disease (PD) can consist of but are not limited to: unequivocal tumour growth of known metastatic lesions, new metastatic lesions, local recurrence.
  - PFS will be determined from serial CT scans, PET-CT or MRT with censoring at the last date alive and progression-free.
- Complete remission, defined as no radiologic sign of residual disease and pathologically complete (R0) resection if applicable.
- Quality of life (QoL) using the EORTC QLQ-C30, QLQ-CR29 and QLQ-LC29 questionnaires at 3, 6, 12, 24 and 36 months after randomisation.

### Participants

Patients with at least 3 technically resectable (R0) pulmonary metastases from colorectal cancer will be enrolled into this trial. A total of 152 patients are planned to be randomised at a 1:1 ratio (about half of the patients to each treatment arm).

#### Main Inclusion Criteria

1. Histologically confirmed colorectal adenocarcinoma
2. ≥ 3 technically resectable (R0) pulmonary metastases
3. Male or female patients aged ≥ 18 years without upper age limit
4. Resected primary tumour with intent to cure (sole prior (chemo) radiation of a rectal cancer with documented complete remission is permitted)
5. In case of previous treatment of hepatic metastases: no radiologic sign of active hepatic disease at the time of trial randomisation
6. A minimum of 12 weeks of systemic therapy with the last treatment applied within 6 months prior to randomisation
7. Good performance status (ECOG 0-1)
8. Sufficient pulmonary reserve (FEV1 >60%, DLCO >60%)

#### Main Exclusion Criteria

1. Active extra-thoracic tumour disease (including primary tumour *in situ*)
2. Prior resection of lung metastases (diagnostic resection is allowed)
3. Requirement of a pneumonectomy to achieve complete resection
4. Other malignancy in the past 5 years (except non-melanoma skin cancer or *in situ* cancer)
5. Histologically proven intrathoracic lymph node metastasis (except resectable single level mediastinal, hilar and pulmonary) as defined at https://radiopaedia.org/articles/thoracic-lymph-node-stations
6. Known or uncontrolled brain metastases
7. Known BRAF V600E mutation (unknown BRAF mutation status does not constitute an exclusion criterion)
8. Prior >2^nd^ line therapy, i.e. TAS-102 (Lonsurf^®^) or regorafenib

9. Medical condition which poses a high risk to undergo systemic treatment and/or surgery as defined by the investigator

### Treatment

All trial participants must have received at least 12 weeks of standard systemic treatment, with the last treatment applied within 6 months prior to randomisation. Chemotherapy can have been carried out at the discretion of the treating oncologist and according to local standards/guideline recommendations and must have consisted of a cytotoxic therapy (monotherapy, doublet or triplet) with or without a VEGF-or EGFR-directed therapy.

If the patients are randomised to Arm A, the pulmonary metastasectomy surgery should be performed as soon as possible after completion of systemic therapy (as defined in inclusion criterion 6) but no sooner than 4 weeks after last application of systemic therapy.

**Figure 1:**
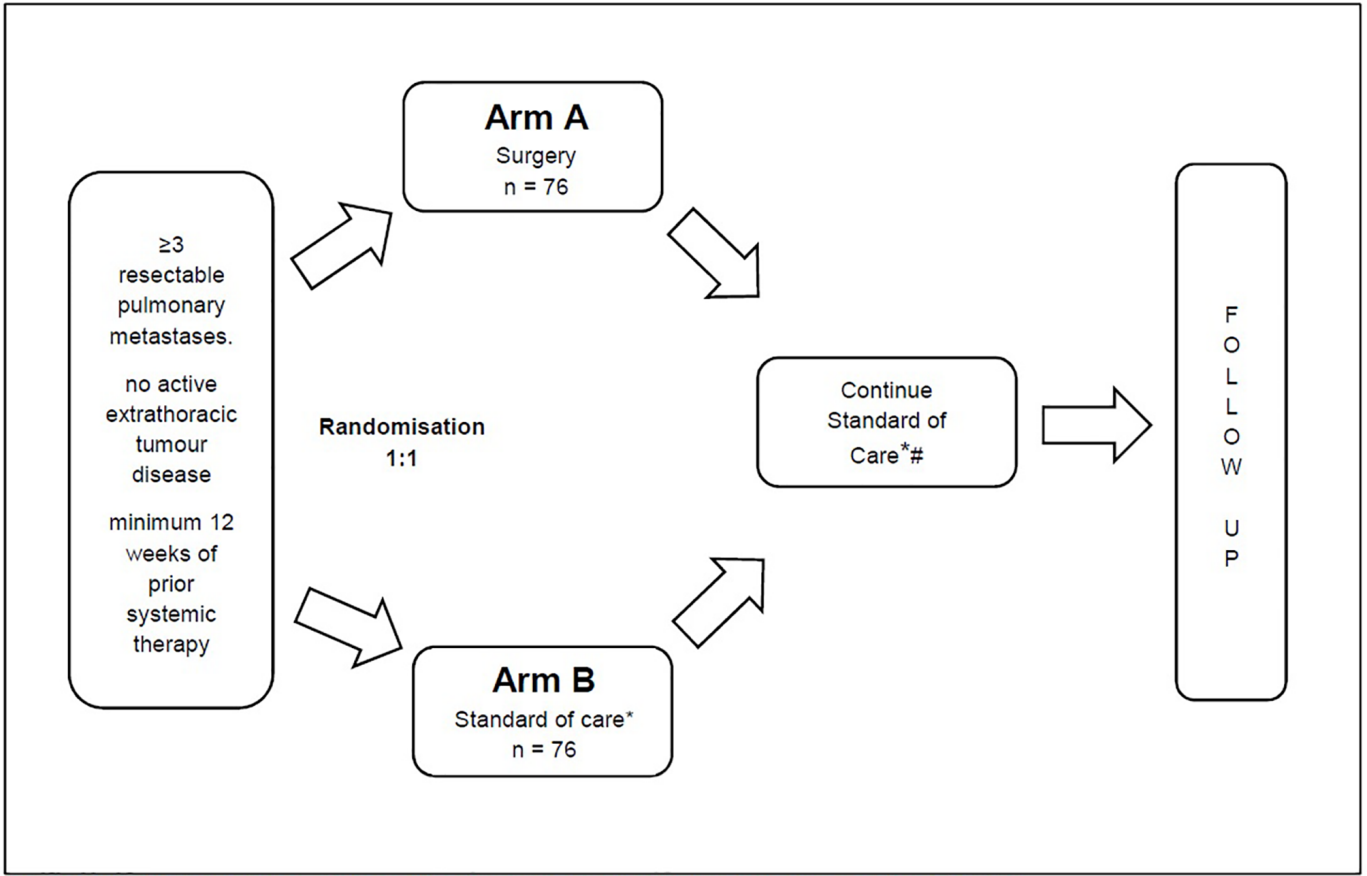
* For Arm B this includes the possibility of local ablative measures such as SBRT. # For Arm A (Surgery) this includes the possibility of re-resection.

Before trial enrolment, all patients will require restaging *via* PET-CT or CT-thorax and -abdomen or CT-thorax plus MRI abdomen within 6 weeks before randomisation. If a patient has 3 or more isolated lung metastases, the patient will be assessed for trial inclusion by an experienced thoracic surgeon, preferably in the setting of a multidisciplinary tumour board. If the metastases are amenable to surgical resection and the inclusion criteria are met, then the patient will be randomised (**Figure 1**).

#### Experimental Treatment (Arm A)

After randomisation into the experimental treatment arm patients will undergo uni- or bilateral surgical resection of the pulmonary metastases.

In case of bilateral disease patients will undergo one side first and after 3-5 weeks the other side. A CT scan of the thorax should be performed after the first surgery and before the second. Remaining metastases after resection on the ipsilateral side do not result in trial exclusion or pose a contraindication to resection of the remaining side. If the lesions are amenable to safe and complete resection, lesions can be removed by the means of minimally invasive surgery. Also, according to surgeon’s preference single-stage bilateral resection *via* sternotomy can be carried out. Single stage bilateral thoracotomy is not recommended. Anatomical resection (segmentectomy, lobectomy) can be applied if it is required to provide safe R0-resection.

Any currently available standard-device can be used for resection according to local standards. If cautery or laser-devices are used the resulting defects should be sutured with a monofilament absorbable suture (e.g. PDS™).

In case of diffuse metastasisation and or pleural carcinosis the surgery must be aborted. Systematic lymphadenectomy or sampling is recommended in patients in Arm A. If mediastinal lymph node metastases are ruled out by PET-CT and/or endobronchial ultrasound guided biopsy (EBUS), lymphadenectomy can be omitted.

After completion of surgical treatment in Arm A the patients will continue with systemic treatment according to the standard of care. Postoperative continuation of systemic therapy will be decided upon investigator’s discretion. If adjuvant treatment is chosen, then this should preferably consist of fluorouracil (alternatively capecitabine) and oxaliplatin. Given the lack of benefit of regimens containing irinotecan, EGFR-targeted agents and VEGF-targeted agents in the adjuvant treatment of stage II or III colon cancer, these should only be used on an individual basis in the postoperative setting.

In the experimental arm, it is encouraged that patients undergo re-resection in case of disease progression/recurrence if the respective oncologic principles apply.

#### Control Treatment (Arm B)

After randomisation into the control arm patients will continue standard of care consisting of systemic therapy. Standard of care should follow common oncologic recommendations. SBRT can be applied upon investigator’s discretion.

**Figure 2:**
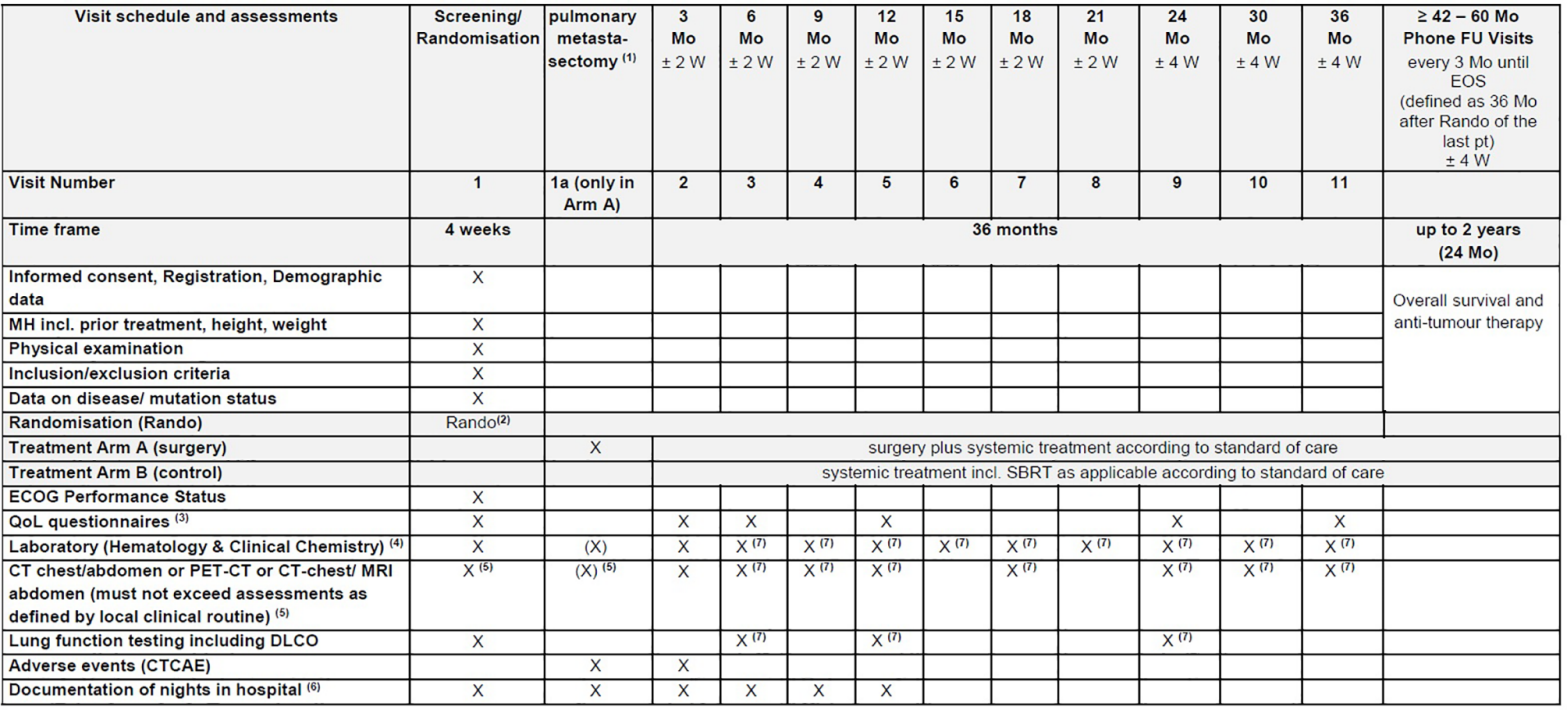
EOS, End of Study; FU, Follow-Up; MH, Medical history; Mo, month(s); pt(s), patient(s); Rando, randomisation; SBRT, Stereotactic Radiation Therapy; W, week(s). (1) Investigations during the treatment period are performed at the discretion of the treating physician and according to the respective treatment arm. (2) Randomisation has to be performed as close as possible to potential start of surgery. (3) Quality of life (QoL) will be assessed using the EORTC QLQ-C30, QLQ-CR29 and QLQ-LC29 questionnaires. (4) Laboratory includes LDH, CEA, CA19-9, CRP (see section 7.8.8 Blood tests). (5) Not older than 6 weeks at the time of randomisation. (6) Number of nights in hospital will be documented starting from the randomisation date and until the end of the month 12. (7) Might be assessed externally, if not possible at trial site due to Covid19.

Data on the standard anti-tumour care (chemotherapy and SBRT) will be collected and analysed **Figure 2**.

### Informed Consent Procedure

Before enrolment in the clinical trial, the patient will be informed that participation in the clinical trial is voluntary and that he/she may withdraw from the clinical trial at any time without having to give reasons and without penalty or loss of benefits to which the patient is otherwise entitled. The treating physician will provide the patient with information about the treatment methods to be compared and the possible risks involved. At the same time, the nature, significance, implications, expected benefits and potential risks of the clinical trial and alternative treatment will be explained to the patient. The patient’s written consent must be obtained before any trial-specific tests/treatments. For this purpose, the written consent form will be personally dated and signed by the trial patient and the investigator conducting the informed consent discussion.

### Randomisation Methodology

Randomisation will be performed, stratified by site, in blocks of variable length aiming for large block lengths in a ratio of 1:1 to ensure a balanced distribution of the treatments and reduce selection bias. The block lengths will be documented separately and will not be disclosed to the sites. Central randomisation will be performed web based using the RedCap™ tool to conceal treatment allocation.

### Data Management and Monitoring

The data management will be performed with REDCap™, a fully web based remote data entry (RDE) system (also called eCRF). The system is based on web forms and is developed and maintained by the REDCap Consortium (redcap@vanderbilt.edu). The technical specifications of the database will be described in the codebook delivered automatically by the REDCap™ system.

Details on data management (software, procedures, responsibilities, etc.) will be described in a data management plan prior to the trial. During the trial, the performance of data management and any deviations from the data management plan will be documented in a data management plan. Technical specifications of the trial data base and all data checks will be documented in a data validation plan.

The trial data base has been fully validated before any data entry will be performed. Data entry personnel will not be given access to the trial data base until they have been trained. The investigator or a designated person will record the participation in the trial, the frequency of the trial visits, the relevant medical data, the concomitant treatment and the occurrence of adverse events in the medical record of each trial patient, as timely as possible. An audit trail provide a data history which data were entered, changed or deleted, by whom and when.

Data will be checked during data entry by so-called built-in edit checks. The data will be further reviewed for completeness, consistency, plausibility, and regarding protocol violations and other distinctive problems (e.g. cumulative missings) using SAS software. The resulting queries will be sent to the investigator for correction or verification of the documented data. All programs which can be used to influence the data or data quality will be validated (e.g. edit check and data validation programs for import of external data, etc.).

Concomitant treatments or procedures entered into the eCRF will be coded using the WHO Drug Reference List. Adverse Events will be coded using the Medical dictionary for regulatory activities (MedDRA) terminology.

Information about trial patients will be kept confidential and managed under the applicable laws and regulations. Those regulations require a signed patient authorisation informing the patient of the following:

· what protected health information (PHI) will be collected from patients in this trial;
· who will have access to that information and why;
· who will use or disclose that information;
· the rights of a research patient to revoke their authorisation for use of their PHI.

In the event that a patient revokes authorisation to collect or use PHI, the investigator, by regulation, retains the ability to use all information collected prior to the revocation of patient authorisation. For patients that have revoked authorisation to collect or use PHI, attempts should be made to obtain permission to collect at least vital status (i.e. that the patient is alive) at the end of their scheduled trial phase. The data collection system for this trial uses built-in security features to prevent unauthorised access to confidential participant information, including an encrypted transport protocol for data transmission from the participating sites to the trial database. The trial database is located on a server of the IT facility of Medical Center - University of Freiburg. Employees of the Clinical Trials Unit charged with hosting the eCRF and the trial database are obliged to maintain data confidentiality and to comply with data protection regulation.

### Biostatistical Planning and Analysis

The sample size calculation is based on the primary endpoint overall survival. A median OS time of approximately 27 months under standard of care is assumed, while the median OS time is expected to increase to about 55 months under standard of care (without SBRT) and pulmonary metastasectomy (2, 21). This corresponds to a hazard ratio of 2.04 between the treatment arms (medical treatment vs. medical treatment and pulmonary metastasectomy). The effect of medical treatment and pulmonary metastasectomy will be assessed by a log-rank test at two-sided significance level of 5% and by estimation of the hazard ratio with corresponding asymptotic two-sided 95% confidence interval. The null hypothesis is rejected, if the confidence interval does not contain one. Under the above assumptions, the trial is planned to detect a difference between medical treatment and pulmonary metastasectomy over medical treatment alone with a power of 90%, which requires a total number of 83 events to be observed. To account for the possibility that the observed hazard ratio may be diminished by non-compliance and/or drop-out of patients, the sample size is calculated to achieve a power of 90%. The required number of patients to be randomised to observe this amount of events depends on the length of follow-up. With a recruitment period of 2 years, an additional follow-up period after the end of recruitment of 3 years (maximum length of follow up 5 years) it can safely be assumed that a sufficient number of events will have been observed by the end of the trial if a total of 152 patients (76 per group) are available for analysis (software used, e.g. nQuery Advisor 8.3).

### Definition of Populations Included in the Analyses

Efficacy analyses will be performed primarily in the full analysis set (FAS) according to the intention-to-treat (ITT) principle. This means that the patients will be analysed in the treatment arms to which they were randomised, irrespective of whether they refused or discontinued the treatment or whether other protocol violations occurred.

The per-protocol (PP) population is a subset of the FAS and is defined as the group of patients who had no major protocol violations, received a predefined minimum dose of the treatment and underwent the examinations required for the assessment of the endpoints at relevant, predefined times. The analysis of the PP population will be performed for the purpose of a sensitivity analysis. Safety analyses will be performed in the safety population. Patients in the safety population are analysed as belonging to the treatment arm defined by treatment received. Patients are included in the respective treatment arm, if treatment was started/if they received at least one dose of trial treatment.

### Primary Endpoint

The effects of standard of care with and without metastasectomy with respect to the primary endpoint overall survival will be estimated and tested by Cox regression. The regression model will include treatment and trial site as independent variables, as well as, metachronicity vs. synchronicity and, previous treatment of hepatic metastasis and colon- or rectal cancer. As estimate of the effect size, the hazard ratio between the two treatment arms will be given with the corresponding asymptotic two-sided 95% confidence interval. The two-sided test on difference between standard of care with metastasectomy and standard of care at significance level 5% will be based on the corresponding asymptotic two-sided 95% confidence interval from the Cox regression model. Overall survival will be analysed irrespective of the occurrence of intercurrent events. This is consistent with the treatment policy strategy of the estimands framework.

### Secondary Endpoints for Efficacy

Descriptive analyses of the secondary endpoints will be performed in similar regression models as for the primary endpoint, as appropriate for the respective type of data. Differences between treatment groups will be calculated with 95% CIs. Progression free survival and complete remission will be measured from randomisation and be analysed using Cox regression as described for the primary endpoint. Endpoints with competing events will be estimated using the Aalen Johanson estimator. Endpoints without competing events with be estimated using the Kaplan Meier estimator. Quality of life measures (EORTC QLQ-C30, QLQ-CR29 and QLQ-LC29) will be analysed descriptively by treatment arm and time point using linear regression. Changes from baseline will be described. Differences in the number of courses of systemic therapy and in the time on systemic therapy will be summarised descriptively by treatment arm using the FAS. The impact of the mutational status on treatment response and survival (on PFS and OS) performed in similar regression models as for primary endpoint adjusted for the mutational status.

## Discussion

Despite its wide application pulmonary metastasectomy in mCRC remains controversial. Past trials have shown ambiguous results which might be at least partially due to difficulties recruiting and/or imbalances in the investigated groups (6, 8, 11). PM are very well tolerated procedures with close to zero mortality and very little morbidity in this often relatively fit patient collective (5, 22). If the situation allows, the treating physician tends to recommend PM to the patient as the common assumption is a prolonged survival when metastases are completely removed. However, as outlined above, this assumption is primarily based on retrospective data, as it has never been formally demonstrated in a prospective trial. Interestingly, one would assume that patients also tend to want to undergo complete removal of metastases, however data from the PulMiCC-trial has shown that patients often seem to prefer non-surgical treatment in case of a well-informed decision (6, 23).

Nevertheless, multiple prospective randomised trials suggest a survival benefit from radical local treatments in oligometastatic cancers (9–11). Hence there is an urgent need for more homogeneous and adequately powered trials in CRC. Considering the discussed issues regarding the heterogeneity in the patient collective, complexity of treatment trajectories, regional differences in treatment choices as well as the aforementioned various biases, well-powered clinical trials have been challenging to design and execute. The goal of this study is to create a setting which allows treatment as close to the real-life conditions as possible but under well standardised conditions. Based on previous trials, in which patient recruitment in the given setting hindered successful study completion, we decided to (1) restrict inclusion to patients with 3 or more metastases (since in case of lesser, surgery will probably be the preferred option) and (2) allow for real world standard of care treatment options before and after randomization including watchful waiting (as opposed to a predefined treatment protocol) and (3) possibility that patient can receive SOC externally (to reduce patient burden). Moreover, we chose to stipulate 12 weeks of systemic treatment prior to possible resection to further standardize treatment response and disease course over a certain period of time. Hence, included patients will be in the disease state of oligopersistence. To increase the feasibility of the trial we took several measures to minimize documentation burden: e.g. certain events are only documented in the experimental arm and radiology data on disease progression is limited to no evidence of disease and evidence of disease, which is then further differentiated into progressive and non-progressive disease. Furthermore, to adequately assess the treatment burden, we included quality of life questionnaires, evaluations regarding the application of chemotherapy, as well as nights spend in hospital. This should reflect the possible negative impact of surgery but also chemotherapy on quality of life during the course of the disease. Finally, novel biomarkers are needed for better risk stratification and identification of patients with high risk for CRC recurrence after surgical metastasectomy, outperforming conventional parameters such as CEA, number of metastases, or the disease-free interval. Therefore, in selected centres circulating DNA (ctDNA) will be analysed at various pre- and post-surgical time points as well as in patients not undergoing surgery to characterize its role as a clinically useful biomarker in patients with mCRC undergoing curative-intent pulmonary metastasectomy within the prospective PUCC trial. With further, well-standardised prospective data we hope to provide stronger evidence for performance of PM and potentially better patient selection.

## Data Availability Statement

The original contributions presented in the study are included in the article/supplementary material. Further inquiries can be directed to the corresponding author.

## Ethics Statement

The studies involving human participants were reviewed and approved by Medical Ethical Committee of Freiburg University Medical Center, No. 21-1612. The patients/participants provided their written informed consent to participate in this study.

## Author Contributions

Conceptualisation: SS, HB, RF, CJ and BP. Project planning: SS, HB, RF, JB, NH, CJ and BP. Writing: SS, HB, CJ. Statistical counseling: CJ. Funding acquisition: SS and RF. Editing: SS, HB, RF, JB, NH, CJ and BP. All authors provided review of the manuscript. All authors read and approved the final manuscript. All authors contributed to the article and approved the submitted version.

## Funding

The PUCC trial is funded by the Deutsche Forschungsgemeinschaft (DFG, German Research Foundation) – project number 418151269. The trial design has been peer-reviewed and approved by the funding body.

## Author Disclaimer

The funder was not involved in the development of the protocol. The funder did not influence the trial design and will not take part in data collection, analysis and interpretation or in writing the manuscript.

## Conflict of Interest

The authors declare that the research was conducted in the absence of any commercial or financial relationships that could be construed as a potential conflict of interest.

## Publisher’s Note

All claims expressed in this article are solely those of the authors and do not necessarily represent those of their affiliated organizations, or those of the publisher, the editors and the reviewers. Any product that may be evaluated in this article, or claim that may be made by its manufacturer, is not guaranteed or endorsed by the publisher.
